# Increased Risk of Non-Alcoholic Steatohepatitis in Patients With Inflammatory Bowel Disease: A Population-Based Study

**DOI:** 10.7759/cureus.35854

**Published:** 2023-03-07

**Authors:** Somtochukwu Onwuzo, Antoine Boustany, Mustafa Saleh, Riya Gupta, Chidera Onwuzo, Jessy Mascarenhas Monteiro, Favour Lawrence, Chukwuemeka Obuekwe, Zoya Morani, Imad Asaad

**Affiliations:** 1 Internal Medicine, Cleveland Clinic Foundation, Cleveland, USA; 2 Faculty of Medical Sciences, Lebanese University, Beirut, LBN; 3 Faculty of Medicine, Kasturba Medical College, Mangalore, IND; 4 Internal Medicine, General Hospital Lagos Island, Lagos, NGA; 5 Internal Medicine, Ross University School of Medicine, Bridgetown, BRB; 6 Internal Medicine, Obafemi Awolowo University Teaching Hospital, Osun, NGA; 7 Medicine, Washington University of Health and Science, San Pedro, BLZ

**Keywords:** population based study, large-database, ulcerative colıtıs, crohn`s disease, non-alcoholic steatohepatitis

## Abstract

Background and objective

The global health burden of inflammatory bowel disease (IBD) stems from its increasing incidence over the years. Comprehensive studies on the topic hypothesize that IBD plays a more dominant in the development of non-alcoholic fatty liver disease (NAFLD) and non-alcoholic steatohepatitis (NASH). In light of this, we conducted this study with the aim of assessing the prevalence and risk factors of developing NASH in patients who have had a diagnosis of ulcerative colitis (UC) and Crohn’s disease (CD).

Methodology

A validated multicenter and research platform database of more than 360 hospitals from 26 different healthcare systems across the United States from 1999 to September 2022 was utilized for conducting this study. Patients aged 18-65 years were included. Pregnant patients and individuals diagnosed with alcohol use disorder were excluded. The risk of developing NASH was calculated using a multivariate regression analysis to account for potential confounding variables including male gender, hyperlipidemia, hypertension, type 2 diabetes mellitus (T2DM), and obesity. A two-sided p-value <0.05 was considered statistically significant, and all statistical analyses were performed using R version 4.0.2 (R Foundation for Statistical Computing, Vienna, Austria, 2008).

Results

A total of 79,346,259 individuals were screened in the database and 46,667,720 were selected for the final analysis based on the inclusion and exclusion criteria. Using multivariate regression analysis, the risk of developing NASH among patients with UC and CD was calculated. The odds of having NASH among patients with UC was 2.37 (95% CI: 2.17-2.60, p<0.001). Similarly, the odds of having NASH were high in patients with CD as well, at 2.79 (95% CI: 2.58-3.02, p<0.001).

Conclusion

Based on our findings, patients with IBD have an increased prevalence and higher odds of developing NASH after controlling for common risk factors. We believe that a complex pathophysiological relationship exists between both disease processes. Further research is required to establish appropriate screening times to enable earlier disease identification and thereby improve patient outcomes.

## Introduction

Non-alcoholic steatohepatitis (NASH) and benign steatosis are on the histologic spectrum of non-alcoholic fatty liver disease (NAFLD), a clinicopathological condition. NASH is defined as hepatic steatosis and inflammation with hepatocyte injury, Mallory hyaline inclusions, and mixed lymphocytic and neutrophilic inflammatory infiltrate in perivenular areas with or without fibrosis [1]. On the other hand, inflammatory bowel disease (IBD) comprises Crohn's disease (CD) and ulcerative Colitis (UC). While CD is characterized by colonic transmural inflammation with skip lesions, UC is a chronic inflammatory condition characterized by relapsing and remitting episodes of inflammation limited to the colon's mucosal layer.

Multiple studies have shown strong associations between IBD and NAFLD. In a study by Bessissow et al., the prevalence of NAFLD was 33.6% in patients with IBD [2]. Also, Sourianarayanane et al. found that NAFLD had an incidence of 8.2% in patients with IBD when compared to patients without NAFLD [3]. In another study by Elchert et al., the overall prevalence of NASH in CD patients was 0.34% compared to 0.08% in the general population [4]. Many possible pathophysiological hypotheses have been proposed to explain this association, including disease-specific risk factors, such as chronic inflammation, steroid exposure, drug-induced hepatotoxicity, malnutrition, and alteration of gut microbiota [5].

NASH is the second leading cause of liver transplantation in the US [6]. Hence, it is critical to identify the risk factors for NASH so that the overall disease burden can be reduced. Our study focuses on understanding the prevalence and risk of developing NASH in patients with IBD by taking into account the confounding factors, including male gender, hyperlipidemia, hypertension, type 2 diabetes mellitus (T2DM), obesity, UC, and CD.

## Materials and methods

Study design

Our cohort’s data were obtained using a validated, multicentered, and daily-updated database called Explorys (Explorys Inc, Cleveland, OH) developed by IBM Watson Health (now known as Merative; Ann Arbor, MI). Explorys consists of electronic health records from 26 different healthcare systems with a total of about 360 hospitals and more than 70 million patients across the United States. Explorys utilizes Systematized Nomenclature of Medicine-Clinical Terms (SNOMED-CT) for the definition of the diseases and pools large outpatient as well as inpatient deidentified data that can be formulated into numerous cohorts according to the clinical element being studied. Explorys does not record individual patient data such as laboratory or imaging results. The approval of the Institutional Review Board was not required since Explorys is a Health Insurance Portability and Accountability Act (HIPAA)-compliant platform. The use of this database has been validated in multiple fields including cardiology, hematology, and gastroenterology.

Patient selection

A cohort of patients with a SNOMED-CT diagnosis of NASH between 1999 and May 2022 was identified. Patients aged 18-65 years were included. Pregnant patients and individuals diagnosed with alcohol use disorder were excluded.

Covariates

Confounding factors associated with NASH were identified and collected if SNOMED-CT diagnoses were available. These were male gender, hyperlipidemia, hypertension, T2DM, and obesity.

Statistical analysis

The prevalence of NASH, UC, and CD was calculated by dividing the respective number of subjects by the total number of subjects in the Explorys database. To account for confounding from the covariates listed above, we conducted 256 searches to explore every probability, with UC and CD representing one of the variables. A univariate analysis was conducted initially for all the variables, followed by a multivariate analysis. Statistical analysis was performed using R version 4.0.2 (R Foundation for Statistical Computing, Vienna, Austria, 2008), and a two-sided p-value <0.05 was considered statistically significant for all analyses. Multivariate analysis was performed to adjust for multiple factors, including male gender, hyperlipidemia, hypertension, T2DM, obesity, UC, and CD.

## Results

A total of 79,346,259 individuals were screened in the database and 46,667,720 were included in the final analysis as per the inclusion and exclusion criteria. There were 34,200 people with NASH with a prevalence rate of 73 per 100,000 (Table 1). The prevalence of NASH was highest among patients with hypertension, hyperlipidemia, and obesity (66%, 64%, and 62% respectively).

**Table 1:** Baseline characteristics of patients with NASH and controls NASH: non-alcoholic steatohepatitis; T2DM: type II diabetes mellitus

Variables	NASH, n (%)	No NASH, n (%)
Smokers	5,640 (16.49)	2,642,640 (5.66)
Male	15,490 (45.29)	21,489,460 (48.08)
Hyperlipidemia	21,880 (63.97)	4,514,960 (9.68)
Hypertension	22,420 (65.55)	5,512,560 (11.82)
T2DM	16,680 (48.77)	2,134,800 (4.57)
Obesity	21,140 (61.81)	3,224,130 (6.91)
Ulcerative colitis	500 (1.46)	118,920 (0.25)
Crohn’s disease	710 (2.07)	161,910 (0.34)
Total	34,200	46,633,520

**Figure 1:**
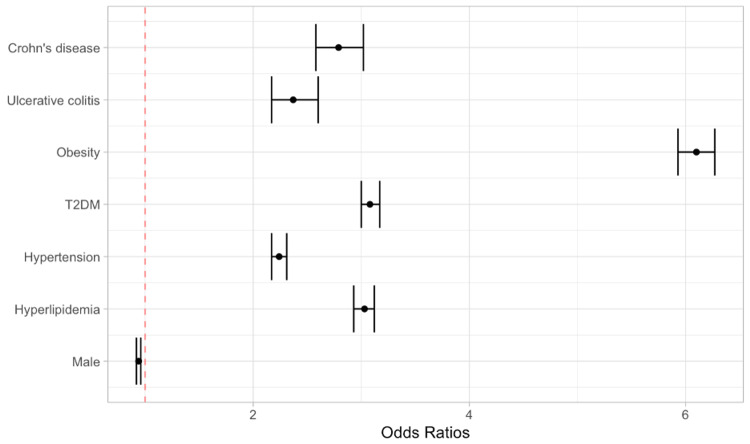
Forrest plot depicting the risk of developing NASH as per stepwise multivariate regression analysis NASH: non-alcoholic steatohepatitis; T2DM: type II diabetes mellitus

Based on multivariate regression analysis, the risk of developing NASH was found to be higher in obese individuals (OR: 6.10; 95% CI: 5.93-6.27), and patients with hyperlipidemia (OR: 3.03; 95% CI: 2.94-3.12), hypertension [odss ratio (OR): 2.24; 95% CI: 2.17-2.60], T2DM (OR: 3.08; 95% CI: 3.00-3.17), UC (OR: 2.37; 95% CI: 2.17-2.60), and CD (OR: 2.79; 95% CI: 2.58-3.02) (Figure 1).

## Discussion

The development of NASH, both in patients with or without a diagnosis of IBD, has been strongly associated with T2DM, hypertension, and metabolic syndrome by multiple lines of evidence [7]. A significant proportion, around 30%, of patients with IBD have been observed to have altered liver inflammatory markers, suggesting the presence of extra-intestinal manifestations in the liver and biliary tract [5]. In fact, primary sclerosing cholangitis (PSC), an autoimmune condition, is observed in 70% of patients with IBD [8]. These findings have led to an increased interest in investigating the association between IBD and NASH. Our study aimed to examine this relationship based on a database of 360 hospitals from 26 healthcare systems in the United States. The results showed that patients with IBD had an increased prevalence of developing NASH compared to those without IBD. The multivariate analysis revealed that individuals with UC (OR: 2.37; 95% CI: 2.17-2.60) and CD (OR: 2.79; 95% CI: 2.58-3.02) were approximately twice as likely to develop NASH compared to those without IBD. This relationship is not as pronounced compared to other risk factors such as obesity (OR: 6.10; 95% CI: 5.93-6.27); however, it remains substantial and comparable to OR associated with T2DM (OR: 3.08; 95% CI: 3.00-3.17), hypertension (OR: 2.24; 95% CI: 2.17-2.60), and hyperlipidemia (OR: 3.03; 95% CI: 2.94-3.12).

The study by Abenavoli et al. (2022) aimed to shed light on the complex interplay between gut microbiota and two increasingly prevalent diseases, NAFLD and IBD [9]. It is worth mentioning that NAFLD encompasses a range of conditions, ranging from simple fatty liver to NASH, which can eventually lead to liver cirrhosis and hepatocellular carcinoma [10]. Several previous studies have demonstrated gut dysbiosis in NAFLD and IBD, each with distinct underlying mechanisms [11-14]. Despite numerous studies supporting a positive association between IBD and NAFLD, a clear and well-established common pathogenesis has yet to be determined, although several emerging hypotheses exist [15]. In effect, they point to a potential interplay between factors such as increased intestinal permeability, changes in gut microbiota, endotoxemia, oxidative stress-induced inflammation, and genetic susceptibility [9,16]. Herein, the overproduction of conjugated bile acids due to gut dysbiosis may contribute to the development of NAFLD when they are returned to the liver [17]. The excessive production of reactive oxygen species (ROS) by Kupffer cells and alteration in mitochondrial DNA demonstrate the slowed progression of NAFLD to NASH, hepatocellular necroinflammation and fibrosis, and lastly carcinoma. Jarmakiewicz-Czaja et al. have also discussed the role of glucocorticoids, a widely used treatment for IBD, as well as other drugs, in developing NAFLD in IBD patients [18]. While investigations into the possible gut-liver immune axis are ongoing, further clinical studies are required to solidify the evidence base for this relationship.

The results of our study endorse previous findings in the literature, notably the most recent ones. Ritaccio et al. conducted a retrospective study of 1,672 IBD patients to examine the prevalence of NAFLD and the progression of NAFLD-related fibrosis [19]. The study found a prevalence of 12.4% for NAFLD, with most patients displaying stable liver fibrosis index at the five-year follow-up. The results were compared to earlier studies from 1992 and 1998, which were limited in terms of their ability to measure the outcomes with the parameters employed adequately. Despite these limitations, the results were consistent with a previous estimate of approximately 20% [20,21]. In a study by Elchert et al., the prevalence of NASH in patients with and without CD was assessed, and the overall prevalence of NASH in CD patients was 0.34% compared to 0.08% in the general population [4]. Magri et al. conducted an observational study involving 178 patients with IBD and found that 40.4% of patients had NASH with varying degrees of steatosis [22]. Another observational study by Principi et al. found a prevalence of 28% of NAFLD in a sample of 465 IBD patients [23]. Lastly, a monocentric cross-sectional study by Hoffmann et al. involving 694 IBD patients found that 48% of patients with CD and 44% of patients with UC had NAFLD, defined by increased echogenicity on liver ultrasound [24]. Despite these concurring results, the influence of the study design employed, sample size, and data analysis on the strength of evidence of these studies still needs to be determined.

A recent study by Yen et al. evaluated the prevalence and risk factors of NAFLD in a retrospective cohort of 81 patients with IBD [25]. Controlled attenuation parameter (CAP) technology with a Fibroscan® was employed for liver stiffness measurement and abdominal ultrasound for the screening program. The study showed that 29.6% of IBD patients were diagnosed with NAFLD based on a CAP value of ≥248 dB/m. However, the results were not statistically significant (p=0.761). The univariate analysis of CD as a risk factor also showed statistically insignificant results (p=0.870). Another study by Mancina et al. employed the same advanced diagnostic liver evaluation using CAP in a cohort of 95 IBD patients and 53 healthy volunteers as a control group [26]. The study's results revealed higher fat content and liver stiffness in IBD patients compared to the control group (CD: 238 ±48 dB/m; UC: 246 ±44 dB/m; controls: 214 ±49 dB/m). The multivariate analysis showed that UC was associated with a higher risk of developing mild steatosis (OR: 4.80; 95% CI: 1.64-14.04) and moderate to severe steatosis (OR: 7.49; 95% CI: 2.36-23.76). These results correlate favorably with the findings of our study that showed a two- to three-fold increased risk of developing NASH.

Several meta-analyses have examined the association between NAFLD and IBD [27,28,29]. The most recent meta-analysis, conducted by Zamani et al., employed a rigorous study design and methodology [29]. The study was based on a systematic search of multiple databases, with the final analysis including 44 studies with 14,947 patients: 19 studies were from Europe, 18 were from the Americas, four were from the Western Pacific, two from South-East Asia, and one from the Middle East. The meta-analysis found that the pooled prevalence of NAFLD in IBD patients was 30.7% (95% CI: 26.5-34.9). A comparison of this result with that of the meta-analysis conducted by Younossi et al. on the global epidemiology of NAFLD in the general population revealed a prevalence of 25.24% (95% CI: 22.10-28.65) [30]. The study further reported that the European region had the highest prevalence of NAFLD among IBD patients (36.9%, 95% CI: 31.2-42.6), while the Eastern Mediterranean region had the lowest (11.8%, 95% CI: 9.7-13.9). The Americas had a prevalence of 28.2% (95% CI: 22.2-34.3), which was consistent with the findings of previous studies. The difference in prevalence observed in our study may be attributed to several factors, including the examination of each IBD disease individually, the exclusion criteria employed, the larger sample size, the diagnostic methods used for the diagnosis of IBD and NAFLD, and the potential impact of different levels of steatosis on the results. Furthermore, the meta-analysis found that IBD patients were 1.96 times more likely to develop NAFLD compared to controls (OR: 1.96; 95% CI: 1.13-3.4), based on a comparison of four studies with a total of 3,884 subjects. The study also found that CD was associated with a 1.16-fold increased risk of NAFLD compared to UC (OR: 1.16; 95% CI: 0.93-1.44).

Individuals diagnosed with IBD are at an elevated risk of readmission and death due to the co-occurrence of NAFLD. They are more vulnerable to metabolic syndrome, given the recent advancements in the treatment of IBD. With the growing global incidence of both NAFLD and IBD, driven by the Westernization of global culture, it is crucial to implement screening protocols to identify IBD patients at risk of NAFLD and use noninvasive methods for early detection of fatty liver. This is particularly important given the findings of our study, which highlight the increased risk of NAFLD in IBD patients in a large cohort.

Our study has several limitations that must be considered. Primarily, other potential confounding factors, such as medication exposure and other chronic liver or biliary diseases, were not considered. In addition, the study did not take into account the different grades of liver steatosis. Furthermore, the study utilized a database from the United States, which limits the generalizability of its results to other populations given the differences in phenotypes of IBD. Finally, the retrospective, population-based design of the study limits our ability to establish a causal relationship between IBD and NASH.

## Conclusions

Multiple studies have indicated an association between IBD and the development of NAFLD. Our study, which examined a database of 360 hospitals in the US, found that individuals with IBD were twice as likely to develop NASH compared to those without IBD. Chronic systemic inflammation seen in patients with IBD, eventually leading to excessive production of ROS and alteration in mitochondrial DNA, is believed to be the underlying link. The underlying mechanisms of this relationship remain unknown, and further research is needed to gain a more comprehensive understanding of the molecular basis of this relationship. This study represents a significant contribution to the field, as it is the first study of its kind with a large sample size and provides a strong foundation for future studies to further explore the causality of this relationship through experimental designs such as randomized controlled trials and large-scale cohort studies.

## References

[1] Antunes C, Azadfard M, Hoilat GJ, Gupta M (2022). Fatty Liver. http://www.ncbi.nlm.nih.gov/books/NBK441992/.

[2] Bessissow T, Le NH, Rollet K, Afif W, Bitton A, Sebastiani G (2016). Incidence and predictors of nonalcoholic fatty liver disease by serum biomarkers in patients with inflammatory bowel disease. Inflamm Bowel Dis.

[3] Sourianarayanane A, Garg G, Smith TH, Butt MI, McCullough AJ, Shen B (2013). Risk factors of non-alcoholic fatty liver disease in patients with inflammatory bowel disease. J Crohns Colitis.

[4] Elchert JA, Mansoor E, Sinh P, Sclair S, Cohen S, Katz J, Cooper G (2018). Prevalence of non-alcoholic steatohepatitis (NASH) in individuals with Crohn's disease: a population-based national study. Am J Gastroenterol.

[5] Rojas-Feria M, Castro M, Suárez E, Ampuero J, Romero-Gómez M (2013). Hepatobiliary manifestations in inflammatory bowel disease: the gut, the drugs and the liver. World J Gastroenterol.

[6] Wong RJ, Singal AK (2020). Trends in liver disease etiology among adults awaiting liver transplantation in the United States, 2014-2019. JAMA Netw Open.

[7] Souza MR, Diniz Mde F, Medeiros-Filho JE, Araújo MS (2012). Metabolic syndrome and risk factors for non-alcoholic fatty liver disease. Arq Gastroenterol.

[8] Wiesner RH, Grambsch PM, Dickson ER (1989). Primary sclerosing cholangitis: natural history, prognostic factors and survival analysis. Hepatology.

[9] Abenavoli L, Giubilei L, Procopio AC, Spagnuolo R, Luzza F, Boccuto L, Scarpellini E (2022). Gut microbiota in non-alcoholic fatty liver disease patients with inflammatory bowel diseases: a complex interplay. Nutrients.

[10] Abenavoli L, Larussa T, Corea A (2021). Dietary polyphenols and non-alcoholic fatty liver disease. Nutrients.

[11] Fujimoto T, Imaeda H, Takahashi K, Kasumi E, Bamba S, Fujiyama Y, Andoh A (2013). Decreased abundance of Faecalibacterium prausnitzii in the gut microbiota of Crohn's disease. J Gastroenterol Hepatol.

[12] Iino C, Endo T, Mikami K (2019). Significant decrease in Faecalibacterium among gut microbiota in nonalcoholic fatty liver disease: a large BMI- and sex-matched population study. Hepatol Int.

[13] Andoh A, Imaeda H, Aomatsu T (2011). Comparison of the fecal microbiota profiles between ulcerative colitis and Crohn's disease using terminal restriction fragment length polymorphism analysis. J Gastroenterol.

[14] Jiang W, Wu N, Wang X (2015). Dysbiosis gut microbiota associated with inflammation and impaired mucosal immune function in intestine of humans with non-alcoholic fatty liver disease. Sci Rep.

[15] Majchrzak K, Dudek P, Talar-Wojnarowska R, Fichna J (2021). Current approach to hepatobiliary manifestations in inflammatory bowel disease. J Physiol Pharmacol.

[16] Mancina RM, Spagnuolo R, Milano M (2016). PNPLA3 148M carriers with inflammatory bowel diseases have higher susceptibility to hepatic steatosis and higher liver enzymes. Inflamm Bowel Dis.

[17] Miao RR, Zhan S, Cui SX, Qu XJ (2022). Intestinal aberrant sphingolipid metabolism shaped-gut microbiome and bile acids metabolome in the development of hepatic steatosis. FASEB J.

[18] Jarmakiewicz-Czaja S, Sokal A, Pardak P, Filip R (2022). Glucocorticosteroids and the risk of NAFLD in inflammatory bowel disease. Can J Gastroenterol Hepatol.

[19] Ritaccio G, Stoleru G, Abutaleb A, Cross RK, Shetty K, Sakiani S, Wong U (2021). Nonalcoholic fatty liver disease is common in IBD patients however progression to hepatic fibrosis by noninvasive markers is rare. Dig Dis Sci.

[20] de Fazio C, Torgano G, de Franchis R, Meucci G, Arrigoni M, Vecchi M (1992). Detection of liver involvement in inflammatory bowel disease by abdominal ultrasound scan. Int J Clin Lab Res.

[21] Riegler G, D'Incà R, Sturniolo GC (1998). Hepatobiliary alterations in patients with inflammatory bowel disease: a multicenter study. Scand J Gastroenterol.

[22] Magrì S, Paduano D, Chicco F (2019). Nonalcoholic fatty liver disease in patients with inflammatory bowel disease: beyond the natural history. World J Gastroenterol.

[23] Principi M, Iannone A, Losurdo G (2018). Nonalcoholic fatty liver disease in inflammatory bowel disease: prevalence and risk factors. Inflamm Bowel Dis.

[24] Hoffmann P, Jung V, Behnisch R, Gauss A (2020). Prevalence and risk factors of nonalcoholic fatty liver disease in patients with inflammatory bowel diseases: a cross-sectional and longitudinal analysis. World J Gastroenterol.

[25] Yen HH, Su PY, Huang SP, Wu L, Hsu TC, Zeng YH, Chen YY (2021). Evaluation of non-alcoholic fatty liver disease in patients with inflammatory bowel disease using controlled attenuation parameter technology: a Taiwanese retrospective cohort study. PLoS One.

[26] Mancina RM, De Bonis D, Pagnotta R (2020). Ulcerative colitis as an independent risk factor for hepatic steatosis. Gastroenterol Nurs.

[27] Zou ZY, Shen B, Fan JG (2019). Systematic review with meta-analysis: epidemiology of nonalcoholic fatty liver disease in patients with inflammatory bowel disease. Inflamm Bowel Dis.

[28] Lin A, Roth H, Anyane-Yeboa A, Rubin DT, Paul S (2021). Prevalence of nonalcoholic fatty liver disease in patients with inflammatory bowel disease: a systematic review and meta-analysis. Inflamm Bowel Dis.

[29] Zamani M, Alizadeh-Tabari S, Singh S, Loomba R (2022). Meta-analysis: prevalence of, and risk factors for, non-alcoholic fatty liver disease in patients with inflammatory bowel disease. Aliment Pharmacol Ther.

[30] Younossi ZM, Koenig AB, Abdelatif D, Fazel Y, Henry L, Wymer M (2016). Global epidemiology of nonalcoholic fatty liver disease-meta-analytic assessment of prevalence, incidence, and outcomes. Hepatology.

